# Prevalence of *Dirofilaria immitis, Ehrlichia canis, Borrelia burgdorferi *sensu lato, *Anaplasma *spp. and *Leishmania infantum *in apparently healthy and CVBD-suspect dogs in Portugal - a national serological study

**DOI:** 10.1186/1756-3305-5-62

**Published:** 2012-03-27

**Authors:** Luís Cardoso, Cláudio Mendão, Luís Madeira de Carvalho

**Affiliations:** 1 Department of Veterinary Sciences, School of Agrarian and Veterinary Sciences, University of Trás-os-Montes e Alto Douro, Vila Real, Portugal; 2 Parasite Disease Group, Instituto de Biologia Molecular e Celular (IBMC), Universidade do Porto, Portugal; 3 Bayer Portugal S.A., Animal Health Division, Carnaxide, Portugal; 4 Interdisciplinary Centre for Research in Animal Health (CIISA), Faculty of Veterinary Medicine, Technical University of Lisbon, Portugal

**Keywords:** *Anaplasma *spp., *Borrelia burgdorferi *sensu lato, Canine Vector-Borne Diseases, Dogs, *Dirofilaria immitis*, *Ehrlichia canis*, Epidemiology, In-Clinic ELISA Tests, *Leishmania infantum*, Portugal

## Abstract

**Background:**

Canine vector-borne diseases (CVBDs) are caused by a wide range of pathogens transmitted to dogs by arthropods including ticks and insects. Many CVBD-agents are of zoonotic concern, with dogs potentially serving as reservoirs and sentinels for human infections. The present study aimed at assessing the seroprevalence of infection with or exposure to *Dirofilaria immitis, Ehrlichia canis, Borrelia burgdorferi *sensu lato, *Anaplasma *spp. and *Leishmania infantum *in dogs in Portugal.

**Methods:**

Based on 120 veterinary medical centres from all the regions of mainland and insular Portugal, 557 apparently healthy and 628 CVBD-suspect dogs were sampled. Serum, plasma or whole blood was tested for qualitative detection of *D. immitis *antigen and antibodies to *E. canis, B. burgdorferi *s. l., *Anaplasma *spp. and *L. infantum *with two commercial in-clinic enzyme-linked immunosorbent assay kits. Odds ratios (OR) were calculated by logistic regression analysis to identify independent risk factors of exposure to the vector-borne agents.

**Results:**

Total positivity levels to *D. immitis, E. canis, B. burgdorferi, Anaplasma *spp., *L. infantum*, one or more agents and mixed agents were 3.6%, 4.1%, 0.2%, 4.5%, 4.3%, 14.0% and 2.0% in the healthy group, and 8.9%, 16.4%, 0.5%, 9.2%, 25.2%, 46.3% and 11.6% in the clinically suspect group, respectively. Non-use of ectoparasiticides was a risk factor for positivity to one or more agents both in the apparently healthy (OR = 2.1) and CVBD-suspect (OR = 1.5) dogs. Seropositivity to *L. infantum *(OR = 7.6), *E. canis *(OR = 4.1) and *D. immitis *(OR = 2.4) were identified as risk factors for the presence of clinical signs compatible with CVBDs. Positivity to mixed agents was not found to be a risk factor for disease.

**Conclusions:**

Dogs in Portugal are at risk of becoming infected with vector-borne pathogens, some of which are of zoonotic concern. CVBDs should be considered by practitioners and prophylactic measures must be put in place to protect dogs and limit the risk of transmission of vector-borne agents to humans. This study is expected to give veterinary and public health authorities an increased awareness about CVBDs in Portugal and to serve as a reference for future investigations and control actions.

## Background

Canine vector-borne diseases (CVBDs) are an emerging problem worldwide due to their frequency and morbidity and, in most cases, also to their zoonotic relevance, with dogs potentially serving as sentinels for human infection [1]. CVBDs are caused by a diverse range of pathogens, mainly bacteria and parasites, which are transmitted to dogs by different arthropod vectors, particularly ticks and insects [2].

Nematode *Dirofilaria immitis*, bacteria *Ehrlichia canis, Borrelia burgdorferi *sensu lato, *Anaplasma phagocytophilum *and *Anaplasma platys*, and protozoan *Leishmania infantum *are among of the major vector-borne agents that can infect dogs [3].

*D. immitis *is transmitted by mosquitoes primarily from genera *Culex, Aedes *and *Anopheles*, and causes dirofilariosis or heartworm disease, a potentially fatal condition in dogs [4]. Canine dirofilariosis is associated with a dry chronic cough, exercise intolerance, dyspnoea, weakness, weight loss, epistaxis, cyanosis and congestive heart failure [5]. Dogs are the natural hosts, but infection may also occur in other canids and cats, and there is also a risk of zoonotic transmission [6]. Human heartworm infections are relatively uncommon; nevertheless, *D. immitis *can cause pulmonary dirofilariosis in people with the occurrence of granulomas in the lungs [7,8].

*E. canis*, a causative agent of acute or chronic canine monocytic ehrlichiosis, is transmitted by the brown dog tick, *Rhipicephalus sanguineus *[9]. Dogs infected with *E. canis *present a spectrum of disease that ranges from subclinical infection to fatal illness [10]. Clinical signs often include lethargy, anorexia, weight loss, hyperthermia, epistaxis and other haemorrhagic disorders, pale mucous membranes and lymph node enlargement [11]. *E. canis *has a zoonotic potential as human infections have been reported from Venezuela [12].

*B. burgdorferi *s.l. spirochetes infect mammals, including dogs and human beings, and cause the so-called Lyme disease [13]. In Europe, *Ixodes ricinus *ticks are important vectors of *B. burgdorferi *s.l. [9]. Most people exposed to *B. burgdorferi *show mild non-specific symptomatology, but Lyme borreliosis can be a chronic debilitating disease in humans, with arthritis, skin changes and neurological or cardiac dysfunction [14]. In contrast, relatively few infected dogs demonstrate clinical signs. However, canine borreliosis has been associated with lethargy, hyperthermia, anorexia, joint inflammation, lameness, lymphadenopathy and glomerulonephritis [15].

*A. phagocytophilum*, the agent of granulocytic anaplasmosis, is vectored in Europe by *I. ricinus *and can infect a wide range of domestic and wild vertebrate hosts, including rodents, horses, dogs and humans [16]. Infection in dogs may be subclinical or result in a mild to severe acute illness, with lethargy, anorexia, hyperthermia, lameness and, occasionally, polydipsia, vomiting, diarrhoea and even neurologic signs [17]. In human beings *A. phagocytophilum *induces a febrile syndrome associated with myalgia and headache, and is considered an emerging pathogen [18].

*A. platys *is a bacterium primarily of dogs that infects platelets and may cause canine infectious cyclic thrombocytopenia [16]. Clinical signs include abnormalities such as lymphadenomegaly and pale mucous membranes, but canine infections with *A. platys *are mostly subclinical [19]. Although its virulence is generally low, *A. platys *might play a role in co-infection with other vector-borne agents [9]. The presumed vector of *A. platys *is *R. sanguineus*.

Dogs are the main reservoir of *L. infantum*, which is transmitted among canines and to humans by phlebotomine sand fly insects, *Phlebotomus *spp. in Europe [20]. Canine leishmaniosis is a systemic chronic condition whose clinical manifestations usually include lymphadenopathy, dermatitis, alopecia, cutaneous ulceration, onychogryphosis, lameness, weight loss, cachexia, ocular lesions, epistaxis, anaemia and renal failure [21]. A large majority of the infected dogs do not develop clinical signs but they may still be capable of transmitting the parasite to the vectors [22]. In people, visceral leishmaniosis is the most severe clinical syndrome resulting from infections with *L. infantum*, and in Europe it is observed mainly in children and immunocompromised adults [23]. Leishmaniosis due to *L. infantum *is a major zoonosis potentially fatal to dogs and humans, and infected dogs represent an important veterinary medical and public health problem [24].

Dogs can be sequentially or simultaneously infected with more than one vector-borne agent by being exposed to arthropods infected with a single pathogen species or to vector(s) concurrently infected with different organisms [2,25]. Some arthropod species, particularly ticks, act as vectors of more than one agent and co-infection of individual arthropods can occur [9]. Awareness of canine co-infections is an important clinical and diagnostic issue as they might induce more severe pathological effects than infections with either agent alone [26].

Diagnosis and screening are essential for the control of CVBDs, both at the individual and population levels, with detection methods including cytological examination of blood smears or other tissues, serology (for antibodies or antigens) and the polymerase chain reaction (PCR). Evidence of infection with or exposure to the causative agents of dirofilariosis, ehrlichiosis, borreliosis, anaplasmosis and leishmaniosis can be assessed via rapid in-clinic serological testing [27]. Results of either single or co-infections must be interpreted in combination with data on the geographical origin, history of vector exposure and clinical status of dogs, along with other confirmatory tests [28].

Environmental changes, especially global warming, have an impact on the arthropods geographical distribution, abundance and vectorial capacity [29]. Together with human and animal population dynamics, including the increased mobility of dogs, climatic changes may affect the occurrence and spread of CVBDs [2]. Updated information on the epidemiology of infection and disease is required to map regional risk, identify new areas of endemicity and forecast CVBD scenarios [30].

*D. immitis, E. canis, B. burgdorferi *s. l., *A. phagocytophilum, A. platys *and *L. infantum *have been detected in Portugal in dogs and/or arthropods [31,32]. Nevertheless, except for the latter [33], there is no comprehensive data available on the regional distribution and prevalence of these vector-borne agents at the countrywide level. The present study aimed at assessing the seroprevalence of infection with or exposure to *D. immitis, E. canis, B. burgdorferi *s. l., *Anaplasma *spp. and *L. infantum *in healthy and CVBD-suspect dogs in Portugal.

## Methods

### Veterinary medical centres and dogs

**Figure 1:**
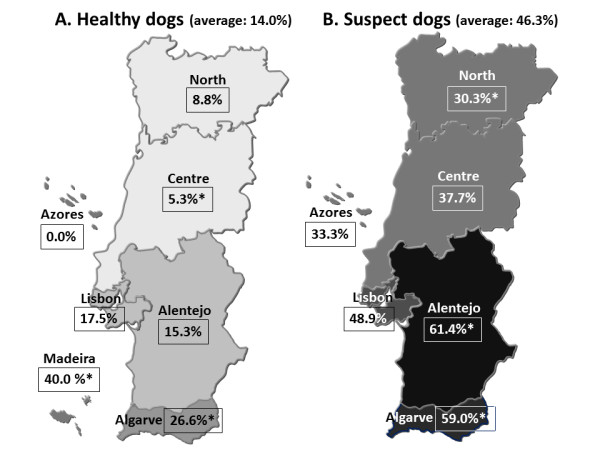
**Positivity to one or more agents in apparently healthy (A) and CVBD-suspect dogs (B) by region**. * Significant difference to the national average. See Tables 1 and 2 for CI for the percentages.

This study was based on a convenience sample of 120 veterinary medical centres from all the Portuguese NUTS (Nomenclature of Units for Territorial Statistics) regions that represented ~15% of the veterinary centres in the country. Point-of-care tests were delivered to 28 centres in the North, 24 in the Centre, 20 in Alentejo, 27 in Lisbon, 19 in the Algarve, one in the Azores and another one in Madeira (Figure 1). Based on a physical examination, local veterinarians were asked to randomly seek for 5 apparently healthy dogs and 5 dogs clinically suspect of a vector-borne disease, including dirofilariosis, ehrlichiosis, borreliosis, anaplasmosis or leishmaniosis.

Suspect or symptomatic dogs had at least one clinical sign of disease, including alopecia, anorexia, cutaneous ulceration, cyanosis, dermatitis, diarrhoea, dry chronic cough, dyspnoea, epistaxis, exercise intolerance, haemorrhagic disorders, hyperthermia, joint inflammation, lameness, lethargy, lymph node enlargement, neurologic signs, ocular lesions, onychogryphosis, pale mucous membranes, polydipsia, vomiting, weakness and weight loss. Healthy or asymptomatic dogs had no signs or historical abnormalities.

From October 2010 to April 2011, 557 apparently healthy dogs and 628 CVBD-suspect dogs were sampled with oral consent from owners. Information was recorded on the animals' gender, age, lifestyle (indoors versus outdoors or mixed), detectable tick infestation, use of ectoparasiticides (acaricides and/or insecticides) and NUTS where they lived. Travel histories were not available.

In the healthy group there were 269 females and 288 males. Median age was 5.0 years (interquartile range [IQR]: 2.0-8.0); age was not recorded for 2 dogs. There were 61 animals aged 12 months or less and 494 older than 12 months. Sixty-five dogs had an indoor lifestyle and 562 had an outdoor or mixed one. There were no detectable ticks in 535 dogs, while 22 had ticks. Ectoparasiticides were used on 371 but not on 168 dogs; no information was available from 18 animals. The numbers of apparently healthy dogs sampled by NUTS are shown in Table 1.

**Table 1:** Prevalence of positivity to vector-borne agents in 557 apparently healthy dogs by Portuguese NUTS region

Region (n)	*D. immitis* (95% CI)	*E. canis* (95% CI)	*B. burgdorferi *s.l. (95% CI)	***Anaplasma *spp**. (95% CI)	*L. infantum* (95% CI)	One or more agents (95% CI)	Mixed agents (95% CI)
North (137)	2.9% (0.8-7.3)	0.7% (0.0-4.0)	0.0% (0.0-2.7)	2.2% (0.5-6.3)	3.6% (1.2-8.3)	8.8% (4.6-14.8)	0.7% (0.0-4.0)
Centre (113)	0.9% (0.0-4.8)	0.9% (0.0-4.8)	0.0% (0.0-3.2)	2.7% (0.6-7.6)	0.9% (0.0-4.8)	5.3%* (2.0-11.2)	0.0% (0.0-3.2)
Alentejo (85)	4.7% (1.3-11.6)	2.4% (0.3-8.2)	1.2% (0.0-6.4)	3.5% (0.7-10.0)	5.9% (1.9-13.2)	15.3% (8.4-24.7)	1.2% (0.0-6.4)
Lisbon (126)	2.4% (0.5-6.8)	6.3% (2.8-12.1)	0.0% (0.0-29)	6.3% (2.8-12.1)	7.9% (3.9-14.1)	17.5% (11.3-25.2)	4.0% (1.3-9.0)
Algarve (79)	5.1% (1.4-12.5)	13.9%* (7.2-23.5)	0.0% (0.0-4.6)	10.1%* (4.5-19.0)	3.8% (0.8-10.7)	26.6%* (17.3-37.7)	5.1% (1.4-12.5)
Azores (7)	0.0% (0.0-41.0)	0.0% (0.0-41.0)	0.0% (0.0-41.0)	0.0% (0.0-41.0)	0.0% (0.0-41.0)	0.0% (0.0-41.0)	0.0% (0.0-41.0)
Madeira (10)	40.0%* (12.1-73.8)	0.0% (0.0-30.8)	0.0% (0.0-30.8)	0.0% (0.0-30.8)	0.0% (0.0-30.8)	40.0%* (12.1-73.8)	0.0% (0.0-30.8)

Total (557)	3.6% (2.2-5.5)	4.1% (2.6-6.1)	0.2% (0.0-1.0)	4.5% (2.9-6.5)	4.3% (2.8-6.3)	14.0% (11.2-17.2)	2.0% (1.0-3.5)

* Statistically significant difference (*p *< 0.05) to the corresponding national average value of prevalence.

Among the CVBD-suspect dogs there were 262 females and 366 males. Median age was 6.0 years (IQR: 3.0-9.0); age was not recorded for 2 animals. Forty-five dogs had 12 months or less and 581 were older than 12 months. Seventy dogs had an indoor lifestyle and 487 had an outdoor or mixed lifestyle; no information was available from one animal. There were no detectable ticks in 577 dogs, while 51 had ticks. Ectoparasiticides were used on 302 but not on 270 dogs; no information was available from 56 animals. The numbers of clinically suspect dogs sampled by NUTS are shown in Table 2.

**Table 2:** Prevalence of positivity to vector-borne agents in 628 CVBD-suspect dogs by Portuguese NUTS region

Region (n)	*D. immitis* (95% CI)	*E. canis* (95% CI)	*B. burgdorferi *s.l. (95% CI)	***Anaplasma *spp**. (95% CI)	*L. infantum* (95% CI)	One or more agents (95% CI)	Mixed agents (95% CI)
North (145)	3.4%* (1.1-7.9)	7.6%* (3.8-13.2)	0.7% (0.0-3.8)	5.5% (2.4-10.6)	18.6% (12.6-25.9)	30.3%* (23.0-38.5)	4.1%* (1.5-8.8)
Centre (122)	7.4% (3.4-13.5)	9.0%* (4.6-15.6)	0.8% (0.0-4.5)	3.3%* (0.9-8.2)	25.4% (18.0-34.1)	37.7% (29.1-46.9)	7.4% (3.4-13.5)
Alentejo (114)	14.0% (8.2-21.8)	25.4%* (17.7-34.4)	0.0% (0.0-3.2)	9.6% (4.9-16.6)	27.2% (19.3-36.3)	61.4%* (51.8-70.4)	13.2% (7.6-20.8)
Lisbon (139)	5.8% (2.5-11.0)	19.4% (13.2-27.0)	0.7% (0.0-3.9)	11.5% (6.7-18.0)	30.2% (22.7-38.6)	48.9% (40.3-57.5)	15.8% (10.2-23.0)
Algarve (105)	17.1%* (10.5-25.7)	23.8% (16.0-31.1)	0.0% (0.0-3.4)	17.1%* (10.5-25.7)	25.7% (17.7-35.2)	59.0%* (49.0-68.5)	20.0%* (12.8-28.9)
Azores (3)	0.0% (0.0-70.8)	0.0% (0.0-70.8)	0.0% (0.0-70.8)	33.3% (0.8-90.6)	0.0% (0.0-70.8)	33.3% (0.8-90.6)	0.0% (0.0-70.8)

Total (628)	8.9% (6.8-11.4)	16.4% (13.6-19.5)	0.5% (0.1-1.4)	9.2% (7.1-11.8)	25.2% (21.8-28.7)	46.3% (42.4-50.3)	11.6% (9.2-14.4)

* Statistically significant difference (*p *< 0.05) to the corresponding national average value of prevalence.

### Testing for serum antigen and antibodies

Serum, plasma or whole blood samples from dogs were screened for simultaneous qualitative detection of circulating *D. immitis *antigen and antibodies, both immunoglobulin G and M, to *E. canis, B. burgdorferi *sensu lato and *Anaplasma *spp. with SNAP^® ^4Dx^® ^test. The same samples were further qualitatively tested for antibodies to *L. infantum *with SNAP^® ^Leishmania. These two rapid tests are commercially available in-clinic enzyme-linked immunosorbent assay (ELISA) kit devices from IDEXX Laboratories (Westbrook, Maine, USA) and were operated according to the manufacturer's instructions listed in the product package insert.

The SNAP^® ^4Dx^® ^*D. immitis *analyte is derived from polyclonal antibodies specific to a carbohydrate antigen of the adult female heartworms [34]. The commercially available in-clinic ELISA detects antibodies against peptides from p30 and p30-1 outer membrane immunodominant proteins of *E. canis *[35]. The *B. burgdorferi *s. l. analyte detects antibodies specific to the C_6 _synthetic peptide derived from the IR6 region within the *Borrelia *membrane protein VlsE [36]. This peptide does not react with antibodies elicited following *B. burgdorferi *vaccination [37]. The *Anaplasma *spp. analyte detects antibodies reacting to a synthetic peptide derived from the immunodominant major outer surface protein (p44/MSP2) of *A. phagocytophilum *[38]. Preliminary studies indicate that the *A. phagocytophilum *analyte in SNAP^® ^4Dx^® ^cross-reacts with samples from *A. platys *infected dogs (SNAP^® ^4Dx^® ^kit insert, unpublished observations) [27]. The antigen used in SNAP^® ^Leishmania is derived from *L. infantum *promastigotes prepared by sonic disruption, filtration and diethylaminoethyl column purification [39].

Reported sensitivities/specificities of the SNAP^® ^4Dx^® ^test are 99.2%/100% for *D. immitis*, 96.2%/100% for *E. canis*, 98.8%/100% for *B. burgdorferi *s. l. and 99.1%/100% for *A. phagocytophilum *[34]. Sensitivity and specificity of SNAP^® ^Leishmania were 91.1/93.4% and 98.3/99.2%, respectively compared with an immunofluorescence antibody test (IFAT) or Western blot [39].

### Data analysis

Exact binomial test was used to calculate confidence intervals (CI) for the proportions, with a 95% confidence level. Chi-square and Fisher's exact tests compared proportions of positivity (no. of dogs found positive divided by the no. of dogs tested) related to categorical dependent variables. Analyses were done with StatLib or SPSS 11.5 software for Windows. A probability (*p*) value < 0.05 was regarded as statistically significant. Binomial variables showing a significant difference between categories were selected to univariate or multivariate logistic regression analysis to identify independent risk factors of exposure to the vector-borne agents, calculating odds ratios (OR) and their 95% CI [40].

## Results

Total and regional positivity of healthy dogs to the several vector-borne agents and to the parameters of one or more agents (≥ 1 agent) and mixed agents (≥ 2 agents) are displayed in Table 1. A map of positivity to one or more agents by NUTS is further presented in Figure 1 (A). Risk factors identified in apparently healthy dogs are shown in Table 3.

**Table 3:** Risk factors for *E. canis, Anaplasma *spp. and one or more agents in apparently healthy dogs

Dependent variable/ /risk factor	*p *value	OR	95% CI
Positivity to *E. canis*			
Positivity to *Anaplasma *spp.	< 0.001	20.8	7.9-55.1
			
Positivity to *Anaplasma *spp.			
Detectable ticks	= 0.185	2.7	0.6-11.4
Non-use of ectoparasiticides	= 0.079	2.3	0.9-5.7
Positivity to *E. canis*	< 0.001	20.7	7.4-58.1
			
Positivity to one or more agents			
Age > 12 months	= 0.040	3.5	1.1-11.5
Non-use of ectoparasiticides	= 0.004	2.1	1.3-3.4

Table 2 displays data on the total and regional positivity of clinically suspect dogs to the vector-borne agents and to the other two parameters. Figure 1 (B) shows a map with positivity to one or more agents by NUTS region. Table 4 presents risk factors identified in CVBD-suspect dogs.

**Table 4:** Risk factors for positivity to vector-borne agents and combined parameters in CVBD-suspect dogs

Dependent variable/ /risk factor	*p *value	OR	95% CI
Positivity to *D. immitis*			
Age > 12 months	= 0.997	1.5^8^	0.0-ND
Outdoor or mixed lifestyle	= 0.073	6.2	0.8-46.1
Positivity to *E. canis*	= 0.036	2.0	1.0-3.7
			
Positivity to *E. canis*			
Age > 12 months	= 0.088	3.5	0.8-15.1
Positivity to *D. immitis*	= 0.085	1.8	0.9-3.5
Positivity to *Anaplasma *spp.	< 0.001	6.1	3.5-10.9
			
Positivity to *Anaplasma *spp.			
Non-use of ectoparasiticides	= 0.011	2.2	1.2-4.2
Positivity to *E. canis*	< 0.001	7.4	4.0-13.5
			
Positivity to *L. infantum*			
Age > 12 months	= 0.030	2.7	1.1-7.4
			
Positivity to one or more agents			
Age > 12 months	< 0.001	4.2	1.9-9.3
Outdoor or mixed lifestyle	= 0.004	2.5	1.3-4.7
Non-use of ectoparasiticides	= 0.022	1.5	1.1-2.1
			
Positivity to mixed agents			
Age > 12 months	< 0.001	4.4	2.0-9.6
Outdoor or mixed lifestyle	= 0.003	2.4	1.4-4.3

ND: not defined.

In decreasing order, seropositivity to *L. infantum *(OR = 7.6, 95% CI: 4.8-11.9; *p *< 0.001), *E. canis *(OR = 4.1, 95% CI: 2.5-6.7; *p *< 0.001) and *D. immitis *(OR = 2.4, 95% CI: 1.4-4.2; *p *= 0.002) were risk factors for the presence of clinical signs compatible with a CVBD. In univariate analysis, positivity to mixed agents was not found to be a risk factor for clinical signs; positivity to one or more agents was not assessed by logistic regression.

Table 5 describes positivity to single and mixed vector-borne agents among the apparently healthy and the clinically suspect dogs. No dog was positive for all agents; 479 healthy (86.0%) and 337 suspect dogs (53.7%) were negative for all five tests.

**Table 5:** Positivity to single and mixed vector-borne agents among 557 apparently healthy and 628 CVBD-suspect dogs

Agents	Healthy (n)	%	Suspect (n)	%
Single agents	67	12.0^a^	218	34.7^a^
				
Di	17	3.1	30	4.8
Ec	14	2.5^b^	47	7.5^b^
Bb	1	0.2	1	0.2
Ap	16	2.9	20	3.2
Li	19	3.4^c^	120	19.1^c^

Mixed agents	11	2.0^d^	73	11.6^d^
				
Di + Ec	0	0.0^e^	6	0.9^e^
Di + Ap	0	0.0	2	0.3
Di + Li	2	0.4	7	1.1
Ec + Bb	0	0.0	1	0.2
Ec + Ap	5	0.9^f^	20	3.1^f^
Ec + Li	0	0.0^g^	17	2.7^g^
Bb + Li	0	0.0	1	0.2
Ap + Li	0	0.0^h^	6	0.9^h^
Di + Ec + Ap	1	0.2	6	0.9
Di + Ec + Li	0	0.0	3	0.5
Di + Ap + Li	0	0.0	1	0.2
Ec + Ap + Li	3	0.5	2	0.3
Di + Ec + Ap + Li	0	0.0	1	0.2

One or more agents	78	14.0^i^	291	46.3^i^

Di: *D. immitis*; Ec: *E. canis*; Bb: *B. burgdorferi *s. l.; Ap: *Anaplasma *spp.; Li: *L. infantum*; ^a-i ^Statistically significant difference (*p *< 0.05).

## Discussion

This is the most comprehensive study carried out in Portugal on the prevalence of infection with or exposure to CVBD-agents regarding the diversity of pathogens and/or the geographical areas under assessment.

In healthy dogs, the highest percentage of *D. immitis *antigen-positive samples was obtained from Madeira (40%), with a significant difference to the national average for heartworm (3.6%; Table 1). The prevalence of *D. immitis *was not assessed in CVBD-suspect dogs from Madeira, but it is predictable that it would surpass the prevalence in the apparently healthy ones. In fact, non-detection of exposure to any other vector-borne agent under assessment strongly suggests that dirofilariosis is the major endemic CVBD in Madeira. In another investigation, Araujo [7] reported a prevalence of canine infection with heartworm microfilariae of 30% in Madeira, 16.5% in Alentejo and 12% in the Algarve. Prevalence may be underestimated if testing is only done for microfilariae and not also for heartworm antigen to reveal occult infection, i.e. adult nematode infection without circulating microfilariae [28,41]. Furthermore, canine filariae *Acanthocheilonema dracunculoides *and *Acanthocheilonema reconditum *exist in Portugal and their microfilariae need to be distinguished from those of *D. immitis *by morphological criteria, staining for acid phosphatase activity or PCR [31,41]. Cross-reactivity with *A. dracunculoides *or *A. reconditum *has not been reported for the heartworm antigen test kits. The rapid ELISA test used in the present study has shown to be highly specific, but sensitivity may decline in dogs with worm burdens of two heartworm females or less [41,42]. Under these circumstances, seropositivity of *D. immitis *antigen may underestimate the true prevalence of infection with the heartworm.

The seroprevalence of antibodies to *E. canis *was significantly higher in the apparently healthy dogs from the Algarve and in the CVBD-suspect ones from Alentejo (Tables 1 and 2). On the other hand, positivity to *E. canis *was significantly lower in the North and the Centre, although it reached regional levels of 7.6% and 9.0%, respectively (Table 2). Molecular identification of *E. canis *by PCR and DNA sequencing has recently been reported in northeastern Portugal [43,44] and in the Algarve [45]. The vector of *E. canis, R. sanguineus*, is the most prevalent tick species of dogs in Portugal and has been found throughout all the regions of the mainland [46]. Seropositivity to *E. canis *was identified as a risk factor for *D. immitis *in the suspect group, probably due to a coincidental higher exposure of *E. canis*-positive dogs to the heartworm (Table 4).

Seroprevalence to *B. burgdorferi *s. l. was the lowest countrywide. Evidence of exposure to the agent of Lyme disease was found in only one healthy dog from Alentejo (Table 1) and in three suspect dogs from the North, the Centre and Lisbon (Table 2). Antibodies to the spirochete were not detected in dogs either apparently healthy or CVBD-suspect from the Azores, the Algarve and Madeira. Nevertheless, DNA of *B. burgdorferi *s. l. (genospecies *B. burgdorferi *sensu stricto, *Borrelia garinii *and *Borrelia afzelii*) has been detected in *I. ricinus *from Madeira [47]. In addition, *B. burgdorferi *s. l. genospecies *Borrelia lusitaniae *was found in *I. ricinus *ticks from a sylvatic habitat in the southern part of the Lisbon region [48]. Other serological studies with IFATs in dogs revealed higher positivity levels in the north-eastern part of Portugal (12.7%) [43] and the Algarve (2.3%) [49]. Differences to the present study may be related with the subpopulations surveyed, which in the study conducted in the Northeast, exclusively comprised of rural and hunting dogs [43].

The seroprevalence of antibodies to *Anaplasma *spp. was significantly higher in the Algarve, for both healthy and suspect dogs, and lower in the Centre, but only for the latter (Tables 1 and 2). Serology has indicated the presence of *A. phagocytophilum *in wild rodents, horses, dogs and human beings in Portugal, although molecular evidence has only been achieved in one seropositive horse [50]. Additionally, two species of *Ixodes *were found to harbour *A. phagocytophilum *DNA: *I. ricinus *from Madeira and *I. ventalloi *from the southern part of Lisbon region [50]. On the other side, *A. platys *infections have been identified by PCR and DNA sequencing in dogs from the North [44] and in *A. phagocytophilum*-seropositive dogs from the Algarve [51]. Serological cross-reactivity between *A. phagocytophilum *and *A. platys *is likely to occur, but sensitivity of the in-clinic ELISA test for the detection of antibodies to *A. platys *still needs to be determined [34]. For specific identification of the canine *Anaplasma *pathogens it is useful to complement serological screening with molecular-based detection methods [38].

*L. infantum*-positive dogs were distributed throughout all the regions in the mainland, with prevalence being significantly higher in the CVBD-suspect animals. Antibodies to the protozoan were not detected in dogs from the Azores or from Madeira. The 4.3% prevalence in the apparently healthy group (Table 1) is statistically similar to the 5.8% seroprevalence from another canine survey, with the DAT (direct agglutination test), in mainland Portugal [33]. In the present study, the percentage of *Leishmania*-positive clinically suspect dogs was higher (*p *< 0.001) than that of any other agent under assessment (Table 2). Seropositivity to *L. infantum *as a single agent was also significantly different between apparently healthy and clinically suspect dogs (Table 5). These findings suggest that *L. infantum*, either as a single or mixed pathogen, was the main cause of illness in dogs, among the vector-borne agents assessed. Nevertheless, they could also indicate that practitioners better recognise the clinical signs of leishmaniosis than those of other CVBDs. In fact, canine leishmaniosis is a common reason why dogs are brought into veterinary centres in Portugal [52].

Although the positivity of test results varied geographically, evidence of current or previous infection with at least one vector-borne agent was found in dogs from all the regions (Tables 1 and 2; Figure 1). Overall, a southerly trend of positivity to *D. immitis, E. canis, Anaplasma *spp., one or more agents and mixed agents was observed both in the apparently healthy and CVBD-suspect dogs. Differences in the regional seroprevalence levels of vector-borne agents are largely determined by the geographical distribution and local density of their arthropod vectors [53]. In the southern regions of the country (Alentejo, Lisbon, the Algarve and Madeira) climatic conditions might be more favourable to the proliferation and abundance of vectors. Nevertheless, there were still considerable levels of infection with or exposure to those agents and their combinations in the North and Centre. Travel histories were not available and the chance of dogs found positive in one region that were infected with or exposed to vector-borne pathogens in another region cannot be excluded.

Age above 12 months, an outdoor or mixed lifestyle and the non-use of ectoparasiticides were identified as risk factors for several dependent variables (Tables 3 and 4). Higher positivity levels for those defined variables are most likely related to an increased cumulative exposure of dogs to arthropod vectors and the agents they transmit. Both apparently healthy and CVBD-suspect dogs which received ectoparasiticides, i.e. commercially available acaricides and/or insecticides, had a significantly lower risk (OR) for positivity to one or more agents. In fact, infections with *E. canis, B. burgdorferi, Anaplasma *spp. and *L. infantum *can be prevented by the direct application on dogs of compounds with acaricidal/insecticidal and anti-feeding properties [54,55]. Besides preventing CVBDs, large spectrum ectoparasiticides also protect dogs from blood loss, local skin disorders and systemic toxicoses potentially caused by arthropod vectors [56]. Prevention of heartworm infection is widely attainable by chemoprophylaxis with macrocyclic lactones [38]. Preventative measures further comprise vaccines against a few CVBDs, including Lyme borreliosis and leishmaniosis [13,20].

Although positive serological results may suggest prior exposure and not necessarily disease, they can alert veterinarians to take into consideration further clinical and diagnostic evaluation of individual dogs [57]. A significantly higher seropositivity to *E. canis *and *L. infantum *as single agents was found among CVBD-suspect dogs (Table 5). In addition, the four combinations of mixed agents with significantly higher seroreactivity in the suspect group included *E. canis, L. infantum *and *D. immitis *(Table 5). Furthermore, positivity to these agents, either as single or mixed pathogens, were identified as risk factors for clinical signs compatible with CVBDs. These observations firmly suggest that leishmaniosis, ehrlichiosis and dirofilariosis should be considered by practitioners in their diagnostic routine. The fact that some CVBD-suspect dogs were negative to the agents assessed raises the hypothesis that they were infected with other vector-borne pathogens, e.g. *Babesia canis, Rickettsia conorii *or *Hepatozoon canis *[25,31].

Many dogs infected with vector-borne agents remain asymptomatic for months or even years, but diagnosis of subclinical infection is important, as those animals might still serve as reservoirs of pathogens to other hosts including humans [2]. In areas of endemicity, an annual serological screening would be recommended to promote early detection and treatment.

## Conclusions

In conclusion, the present study provides evidence that dogs in Portugal are at risk of becoming infected with vector-borne pathogens, some of which are of zoonotic concern. Although exposure can vary according to agent and geographical region, the likelihood of infection with at least one agent is considerable countrywide. In effect, CVBDs caused by single or mixed agents should be taken into account in the clinical management of canine patients. Moreover, prophylactic measures, including the use of ectoparasiticides against arthropods, must be put in place in order to protect dogs and simultaneously limit the risk of zoonotic transmission of vector-borne pathogens. Finally, this study is expected to give veterinary and public health authorities an increased awareness about the picture of CVBDs in Portugal with a view to establishing future control programs.

## Competing interests

The authors declare that they have no competing interests.

## Authors' contributions

CM conceived, designed and coordinated the field study; LC and LMdC participated in the study design and drafted the manuscript. All authors read and approved the final manuscript.
